# Induction and Regeneration of Microspore-Derived Embryos for Doubled Haploid Production in Cabbage (*Brassica oleracea* var. *capitata*)

**DOI:** 10.3390/plants15020221

**Published:** 2026-01-10

**Authors:** Su Bin Choi, Suk Yeon Mo, Han Yong Park

**Affiliations:** Department of Bioresources Engineering, Sejong University, Seoul 05006, Republic of Korea; choisubin98@naver.com (S.B.C.); ysmo0708@naver.com (S.Y.M.)

**Keywords:** green cabbage (*Brassica oleracea* var. *capitata)*, microspore culture, embryogenesis, plant regeneration, flow cytometry, simple sequence repeats

## Abstract

Cabbage (*Brassica oleracea* L. var. *capitata*) is an important leafy vegetable crop, and the development of homozygous parental lines is essential for F_1_ hybrid breeding. Isolated microspore culture (IMC) provides a rapid approach for producing haploid and doubled haploid (DH) lines. However, its efficiency in cabbage remains highly dependent on genotype, donor plant growth conditions, and culture conditions. This study aimed to optimize key factors affecting microspore embryogenesis and plant regeneration in a Korean green cabbage (‘SJ-Ca 13’) and to evaluate the ploidy and genetic characteristics of regenerated plants. Microspore yield and embryogenesis were strongly influenced by flower bud size. Bud size of 4.0 ± 0.5 mm yielded the highest number of microspores (4.17 × 10^4^ per bud) and exclusively produced microspore-derived embryos (2.33 embryos per Petri dish), whereas smaller or larger buds failed to induce embryogenesis. Heat shock treatment at 32.5 °C was essential for embryogenesis, with 24 or 48 h of treatment inducing embryo formation, while prolonged exposure (72 h) completely inhibited embryogenesis. Efficient shoot regeneration was achieved when microspore-derived embryos were cultured on semi-solid MS medium with reduced salt strength (1/3×) and higher agar concentration (1.0%), resulting in the highest shoot regeneration rate. Ploidy test revealed that 50% of regenerated plants were spontaneous doubled haploids. SSR analysis using 26 markers detected no genetic polymorphism among regenerated plants. Overall, this study establishes an efficient IMC and regeneration system for cabbage and demonstrates its potential for rapid DH line production to support cabbage breeding programs.

## 1. Introduction

Cabbage (*Brassica oleracea* L. var. *capitata*) is a leafy vegetable belonging to the Brassicaceae family, containing various bioactive compounds such as glucosinolates, anthocyanins, and amino acids [1]. Extracts from cabbage exhibit efficacy in inhibiting cancer cell proliferation [2]. As these anti-cancer and antioxidant effects become known, cabbage consumption is trending upward. Recently, Korea’s cabbage cultivation area reached approximately 8347 ha, with production at 340,852 tons, representing increases of 29.7% and 30%, respectively, compared to the previous year [3].

Currently, most commercial cabbage cultivars are F_1_ hybrids, which require parental inbred lines with desirable traits. However, traditional breeding methods for developing inbred lines require 7–8 years and considerable time and labor. In contrast, isolated microspore culture (IMC) enables the production of homozygous lines within only 1–2 years [4]. In this technique, although microspores normally follow the gametophytic pathway to develop into pollen, an appropriate combination of culture conditions and stress factors can reprogram their development to follow the sporophytic pathway. As a result, microspore-derived embryos are formed, which are subsequently developed into haploid or doubled haploid (DH) plants [5].

Since Lichter (1982) [6] first reported the successful application of microspore culture in rapeseed (*Brassica napus*), the basic rapeseed microspore culture protocol developed by Pechan and Keller (1988) [7] has served as the foundation for doubled haploid (DH) technology in *Brassica* crops. Based on this protocol, microspore culture has been applied to various *Brassica* species, including broccoli (*Brassica oleracea* var. *italica*) [8], kale (*Brassica oleracea* var. *acephala*) [9], and pak choi (*Brassica rapa* subsp. *chinensis*) [10], and numerous studies have been conducted to improve protocols for microspore embryogenesis. Microspore culture in cabbage (*Brassica oleracea* var. *capitata*) was first attempted by Cao et al. (1994) [11], and subsequent studies have focused on the production of DH plants under various culture conditions [12,13]. In Korea, although studies on cabbage microspore embryogenesis in relation to culture duration and conditions have been reported [14], recent studies remain limited.

In microspore culture, factors such as donor plant growth conditions, bud size, heat shock, culture density and medium composition function together as key determinants of embryogenesis. Previous studies have reported that microspore viability and the frequency of microspore-derived embryo formation are associated with bud size, which is commonly used as an indirect readout of the underlying chromatin status, and that the selection of appropriate bud size provides microspores at the most responsive developmental stage, thereby enhancing the success rate of microspore culture [7]. Heat shock is an effective stress factor that induces isolated microspores to switch toward embryogenic development and plays an essential role in *Brassica* microspore culture. However, the optimal temperature and treatment duration may vary by genotype [12].

In microspore culture, the frequency of normal plantlet regeneration is generally very low, and most plantlets are regenerated indirectly through adventitious shoot formation from swollen embryos or callus [15]. In particular, embryos obtained from microspore culture in Brassicaceae exhibit a markedly low conversion rate into normal plants, which is a major limitation of microspore culture technology. According to Yuan et al. (2010) [16], the regeneration rate was higher on B5 medium than on MS medium, and a regeneration rate of 25% was achieved on medium with 1.0% agar. However, these conditions may not be universally optimal for regeneration. More recently, studies on white cabbage have reported successful plant regeneration from microspore-derived embryos on 1× MS medium (0.7% agar) supplemented with various plant growth regulators [17].

Microspore culture is an efficient approach for the rapid production of haploid and doubled haploid plants, thereby accelerating breeding programs. However, microspore-derived regenerants frequently exhibit variable ploidy levels due to spontaneous chromosome doubling or mixoploidy during in vitro culture, making ploidy assessment an essential step in doubled haploid technology. Flow cytometry provides a rapid and reliable means of ploidy determination by estimating nuclear DNA content from a small amount of plant tissue using DNA-specific fluorescent dyes, and is widely regarded as the most efficient alternative to labor-intensive chromosome counting [18,19].

SSR markers are highly polymorphic, co-dominant, and somatically stable, making them well suited for assessing genetic uniformity and heterozygosity. In microspore-derived plants, SSR analysis has been widely applied to distinguish gametophytic from somatic origin and to confirm homozygosity in doubled haploid lines across various crop species [20,21,22]. Thus, SSR markers provide a reliable molecular approach for verifying the genetic integrity of microspore-derived regenerants [23].

Several studies have been reported mainly on white cabbage; however, research on microspore culture in green cabbage is limited. Therefore, this study aimed to evaluate the effects of bud size, heat shock duration, and regeneration media on the efficiency of isolated microspore culture in Korean green cabbage ‘SJ-Ca 13’, and to obtain DH lines through ploidy analysis.

## 2. Results

### 2.1. Microspore Yield, Development Stage, and Embryogenesis in Relation to Bud Size

**Figure 1:**
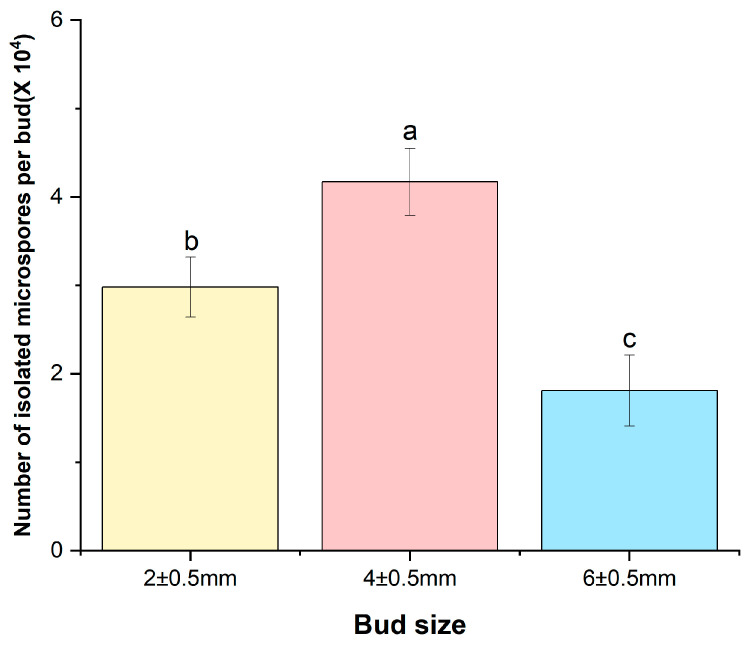
Number of isolated microspores per bud (×10^4^) according to bud size in green cabbage ‘SJ-Ca 13’. Data are presented as mean  ±  standard deviation (SD) (n  =  3). Different lower-case letters on bars indicate statistically significant intergroup differences (*p*  <  0.05).

After microspore isolation, the number of microspores released per flower bud was counted using a hemocytometer according to bud size. The bud size of 4 ± 0.5 mm yielded the highest mean number of microspores (4.17 × 10^4^ per bud), whereas bud sizes 2 ± 0.5 mm and 6 ± 0.5 mm yielded lower numbers, with mean values of 2.98 × 10^4^ and 1.81 × 10^4^ microspores per bud, respectively. These differences were statistically significant among the bud sizes (Figure 1).

**Figure 2:**
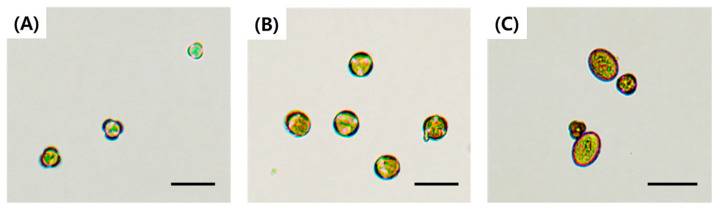
Developmental stages of microspores and young pollen immediately after isolation according to bud size in green cabbage ‘SJ-Ca 13’. (**A**) 2 ± 0.5 mm; (**B**) 4 ± 0.5 mm; (**C**) 6 ± 0.5 mm (young pollen). Scale bar = 40 µm.

Cytological observation of microspores revealed clear differences among flower bud sizes. In bud size of 2 ± 0.5 mm, microspores were commonly observed to be small, relatively transparent, and characterized by three distinct lobes. In contrast, the bud size of 4 ± 0.5 mm contained mostly microspores at the mid–late uninucleate stage, in which the boundaries between the three lobes were partially relaxed. In buds measuring 6 ± 0.5 mm, oval-shaped microspores with rough surface textures were mainly observed (Figure 2).

**Figure 3:**
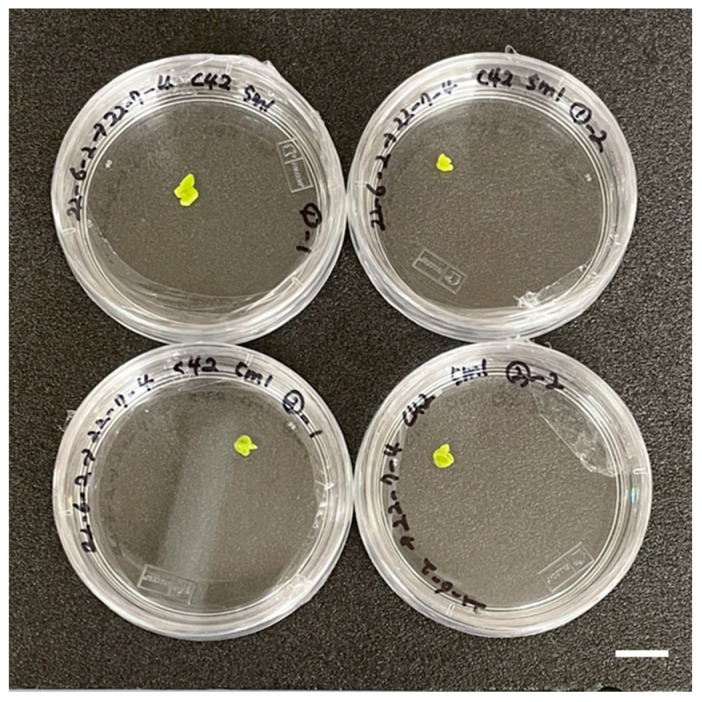
Microspore-derived embryo obtained from 4 ± 0.5 mm buds after 4 weeks of microspore culture in green cabbage ‘SJ-Ca 13’. Scale bar = 1 cm.

Microspore-derived embryos were obtained only from buds measuring 4 ± 0.5 mm, with an average of 2.33 embryos per Petri dish. In contrast, no embryogenesis was observed from bud size of 2 ± 0.5 mm or 6 ± 0.5 mm (Table 1, Figure 3). These results confirm that microspore embryogenesis was induced exclusively from microspores at a specific developmental stage, corresponding to flower buds of 4 ± 0.5 mm in length.

**Figure 4:**
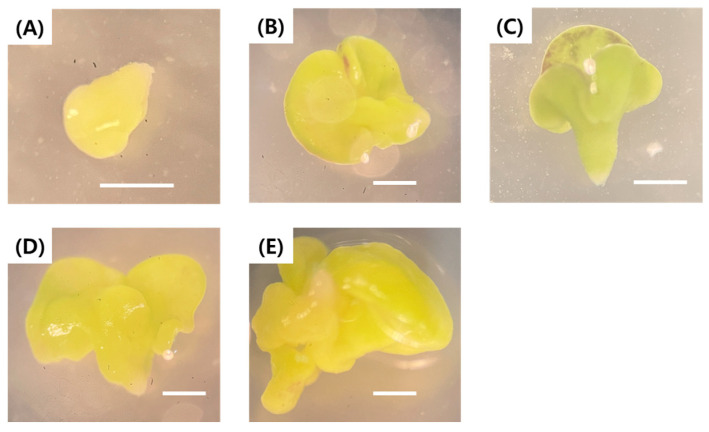
Morphological development of microspore-derived embryos in green cabbage ‘SJ-Ca 13’. (**A**) Torpedo-stage embryo; (**B**,**C**) Cotyledonary-stage; (**D**,**E**) cotyledonary-stage embryos showing abnormal cotyledon development. Scale bar = 1 mm.

When the embryos became visible to the naked eye, the Petri dishes were transferred from dark to light conditions for further development. After 30 days of culture, embryos had developed to the torpedo stage. After 35 days of culture, embryos reached the cotyledonary stage, and morphological variation was observed among embryos. In all embryos, the cotyledons were enlarged relative to the hypocotyl and were poorly separated (Figure 4), likely due to insufficient hormone canalization and transport.

### 2.2. Embryogenesis in Relation to Heat Shock Duration

**Figure 5:**
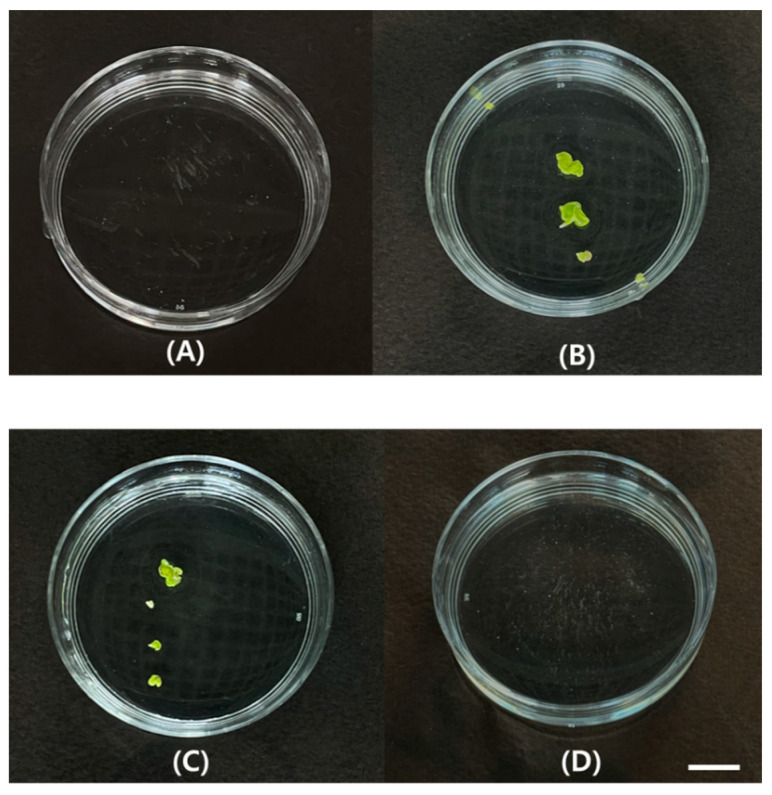
Microspore embryogenesis under different heat shock durations in green cabbage ‘SJ-Ca 13’. (**A**) 0 h; (**B**) 24 h; (**C**) 48 h; (**D**) 72 h. Scale bar = 1 cm.

Heat shock treatment at 32.5 °C was essential for the induction of embryogenesis in microspore cultures of the green cabbage ‘SJ-Ca 13’. The number of embryos formed per Petri dish varied with the duration of heat shock treatment, averaging 2.33 ± 0.52 and 2.00 ± 0.82 embryos after 24 h and 48 h of treatment, respectively. However, no embryogenesis was observed after 72 h of heat shock treatment (0.00 ± 0.00 embryos per Petri dish), indicating complete inhibition of microspore embryogenesis by prolonged heat exposure (Table 2, Figure 5).

### 2.3. Regeneration in Relation to MS Salt Strength and Agar Concentration of Medium

The effects of agar concentration and salt strength of MS semi-solid media on shoot regeneration from microspore-derived embryos were evaluated. Under 0.9% agar, shoot regeneration was observed only in 1/3× MS medium, with a shoot regeneration rate of 16%, whereas no regeneration occurred in 1/2× or 1× MS medium. In contrast, increasing the agar concentration to 1.0% resulted in shoot regeneration rates of 8% in 1/2× MS and 28% in 1/3× MS. Notably, embryos cultured on 1/3× MS medium supplemented with 1.0% agar produced the highest mean number of shoots per embryo (2.4) (Table 3).

**Figure 6:**
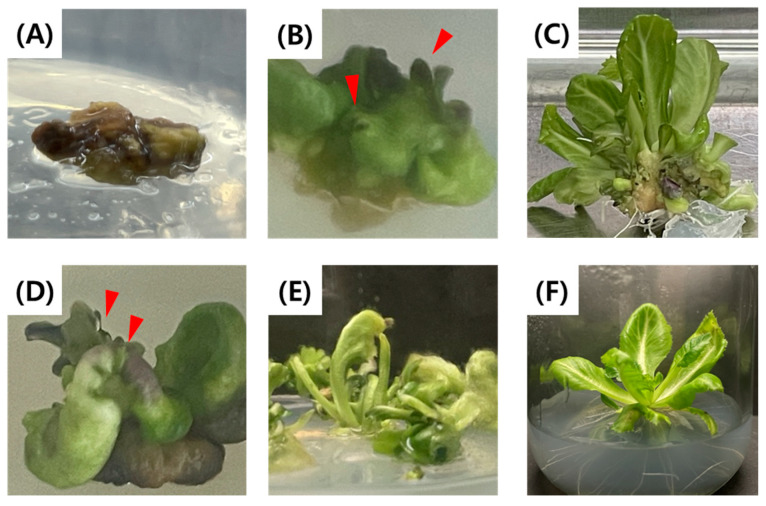
Regeneration patterns of microspore-derived embryos in green cabbage on solid MS culture medium. (**A**) Browning of microspore-derived embryos on 1× MS with 0.9% agar; (**B**) Emergence of shoot-like structures from microspore-derived embryos on 1/3× MS with 1.0% agar, (**C**) Adventitious shoot formation from microspore-derived embryos on 1/3× MS medium with 0.9% agar; (**D**) Emergence of shoot-like structures from microspore-derived embryos on 1/2× MS medium with 1.0% agar; (**E**) Adventitious shoot formation from microspore-derived embryos on 1/2× MS medium with 1.0% agar; (**F**) Fully regenerated plantlet. The red arrows indicate shoot formation.

Microspore-derived embryos cultured on 1× MS semi-solid medium containing 0.9% agar exhibited browning and subsequently died within four weeks of culture (Figure 6A). By contrast, reducing the salt strength to 1/3× MS and increasing the agar concentration to 1.0% induced direct shoot bud formation from embryos (Figure 6B). Multiple adventitious shoots (Figure 6C) developed from the shoot buds were subsequently separated and subcultured to obtain normal plantlets. On 1/2× MS medium with 1.0% agar, direct germination was observed in some embryos (Figure 6D). However, some adventitious shoots showed abnormal morphology (Figure 6E). Following subculture, normal growth was restored in some of the regenerated shoots (Figure 6F).

Overall, efficient regeneration of microspore-derived cabbage embryos was obtained when low MS salt strength (1/3×) was combined with a higher agar concentration (1.0%).

### 2.4. Acclimatization

**Figure 7:**
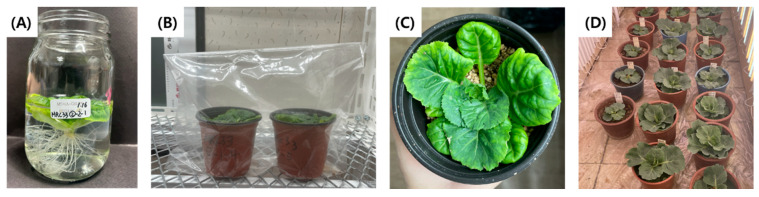
Acclimatization of regenerated plantlets from microspore-derived embryos in green cabbage ‘SJ-Ca 13’. (**A**) Removal of medium from plantlets and root washing with lukewarm water before transplantation; (**B**) Transplantation of regenerated plantlets into 3.5-inch pots of sterilized mixture soil containing perlite:vermiculite (2:1), covered with a plastic bag; (**C**) Regenerated plantlets after humidity acclimation with the plastic cover removed; (**D**) Transplantation of regenerated plantlets into pots containing 100% commercial horticultural soil.

Regenerated plantlets from microspore-derived embryos of the green cabbage ‘SJ-Ca 13’ were successfully acclimatized and transplanted to soil through a stepwise acclimatization process (Figure 7). All regenerated plants survived throughout the acclimatization stages, resulting in a final survival rate of 100%.

### 2.5. Ploidy Level Determination of Regenerated Plants

**Figure 8:**
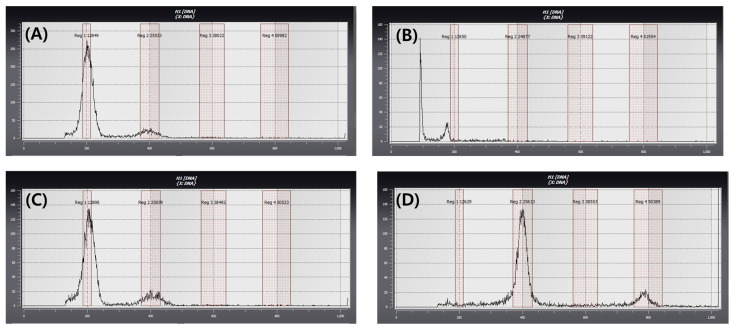
Flow cytometry analysis of regenerated plants from microspore-derived embryos in green cabbage ‘SJ-Ca 13’. (**A**) Control (Diploid, 2n); (**B**) Haploid (n); (**C**) Spontaneous doubled haploid (2n); (**D**) Tetraploid (4n).

After acclimatization, the ploidy levels of regenerated plants from microspore culture were determined by flow cytometry using young leaves, with the fluorescence peak of the donor plant fixed at 200 as a reference (Figure 8). Regenerated plants showing fluorescence peaks at 100, 200, and 400 were classified as haploid (n), spontaneous doubled haploid (2n), and tetraploid (4n), respectively. Among the regenerated plants, 50% were identified as spontaneous doubled haploids (2n), while haploid (n) and tetraploid (4n) plants each accounted for 25%, corresponding to three plants per ploidy level (Table 4).

**Figure 9:**
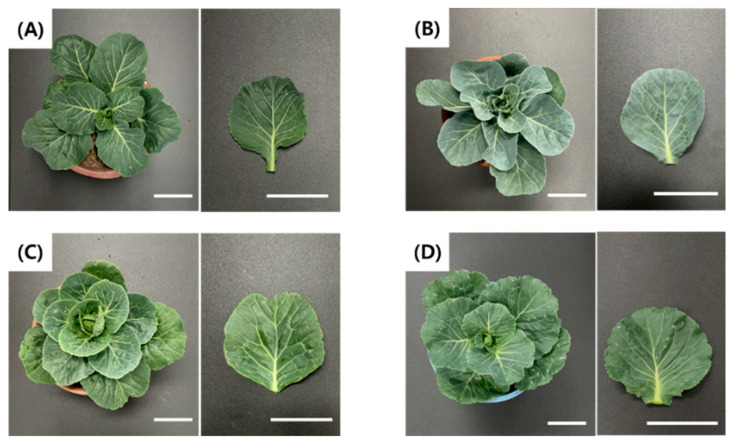
Morphological observation of regenerated plants from microspore-derived embryos in green cabbage ‘SJ-Ca 13’ after two months of acclimatization with different ploidy levels. (**A**) Control (2n); (**B**) Haploid (n); (**C**) Spontaneous doubled haploid (2n); (**D**) Tetraploid (4n). Scale bar = 10 cm.

After ploidy determination by flow cytometry, regenerated plants were grown for two months and evaluated for plant width and leaf length. Compared with the control (SJ-Ca 13, 2n), all regenerated plants exhibited reduced growth. Although the mean plant width and leaf length tended to increase with increasing ploidy level, these differences were not statistically significant (Table 5). Leaf shape and morphological characteristics varied among regenerated plants (Figure 9).

**Figure 10:**
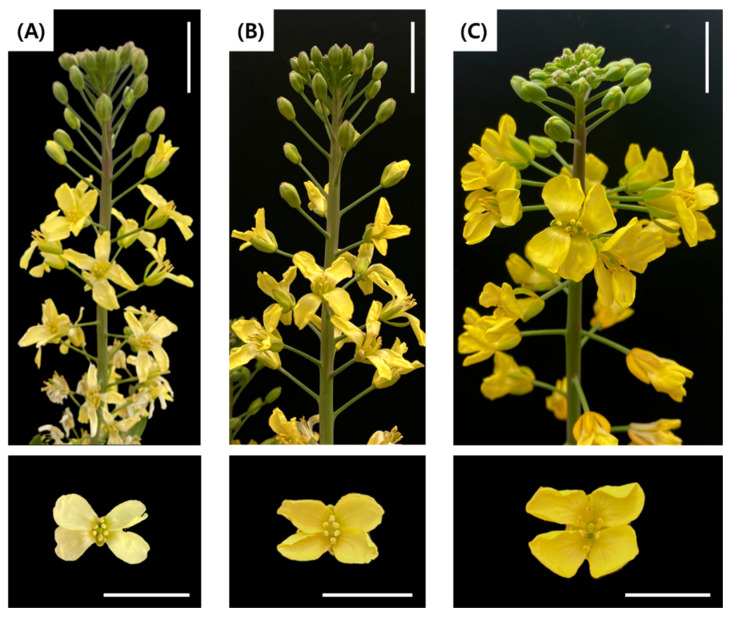
Comparison of floral morphology between a spontaneously doubled plant and a tetraploid plant from microspore-derived embryos in green cabbage ‘SJ-Ca 13’. (**A**) Donor plant (control, 2n); (**B**) Spontaneously doubled haploid (2n); (**C**) Tetraploid (4n). Scale bar = 2 cm.

Regenerated plants identified as haploids failed to undergo bolting and flowering, preventing observation of floral morphology. Therefore, floral characteristics of spontaneous doubled haploid (2n) and tetraploid (4n) regenerated plants were compared with those of the donor plant (2n). The flowers of spontaneous doubled haploid plants (Figure 10B) showed normal growth and pollen formation comparable to the donor plant (Figure 10A), with normal stamen and pistil structures. In contrast, tetraploid plants developed larger flowers and flower buds than the donor plant (2n) and produced a greater amount of pollen, while all floral organs appeared morphologically normal (Figure 10C).

### 2.6. SSR Marker Analysis of Regenerated Plants

**Figure 11:**
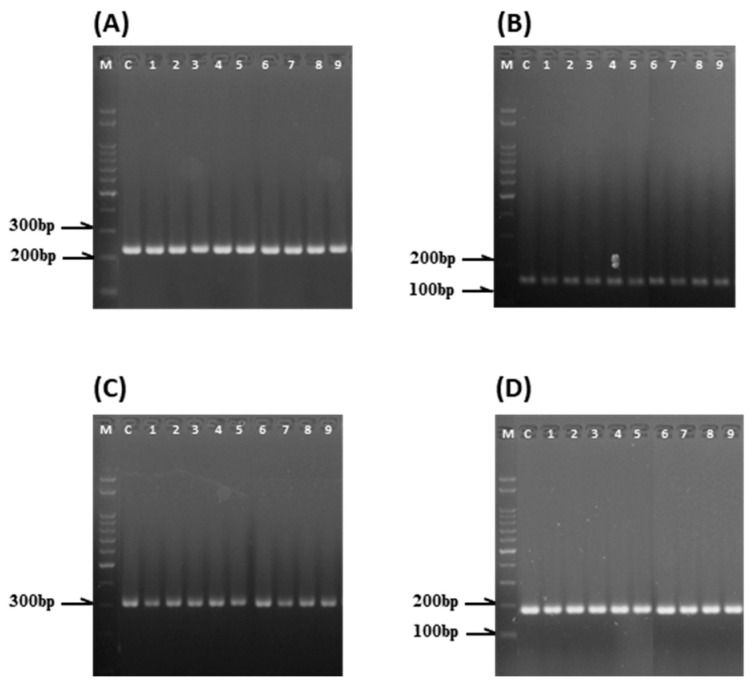
PCR amplification profiles of SSR markers ((**A**): BoL-5, (**B**): BoL-12, (**C**): BoL-14, (**D**): BoL-18) in regenerated plants from microspore-derived embryos of cabbage ‘SJ-Ca 13’. M, 100 bp size marker; C, donor plant ‘SJ-Ca 13’; lanes 1–3, tetraploid plants; lanes 4–9, spontaneously doubled haploid plants.

Analysis of microspore-derived plants using 26 SSR markers routinely applied for cabbage seed purity testing revealed that all tetraploid (4n; n = 3) and spontaneous diploid (2n; n = 6) plants exhibited monomorphic banding profiles identical to those of the donor plant (2n). Consistently, no SSR polymorphism was detected among the regenerated plants (Figure 11). Thus, the microspore-derived plant population of ‘SJ-Ca 13’ showed no genetic diversity within the SSR markers used in this study.

## 3. Discussion

The impact of flower bud size on microspore yield is significant and varies not only across species but also among genotypes within species. This variation can largely be attributed to differences in the physiological development rate and the developmental stages of microspores among these genotypes. To optimize microspore culture, it is crucial to understand the relationship between bud size and microspore developmental stage with in a given genotype. This includes assessing the proportion of microspores that are at the appropriate developmental stage. By incorporating these factors into the experimental design, the efficiency of microspore culture can be enhanced, ultimately improving embryogenesis.

In this study, the highest mean number of microspores (4.17 × 10^4^ per bud) and successful embryogenesis were achieved from microspores obtained from buds sized at 4 ± 0.5 mm. This result aligns with findings in *Brassica napus* and *B. juncea* in which high embryogenesis efficiency has been reported in bud size of 3–4 mm, which mainly contains microspores at the mid- to late-uninucleate stage [24]. In the case of white cabbage, the highest embryogenic responsiveness was observed in buds of 4.0–5.0 mm, corresponding to the late uninucleate to early binucleate stage [25]. This suggests a consistent trend across different species that specific bud sizes are optimal for microspore development and subsequent embryogenesis. Further supporting this, in cauliflower cultivars ‘Pusa Kartik Sankar’ and ‘Pusa Sharad’, optimal embryogenesis was observed in buds of 4.0–4.5 mm [26], whereas in five genotypes of mustard (*Brassica carinata*), the highest number of embryos was obtained from buds measuring 2.5–3.5 mm [27]. Moreover, the impact of bud size on both microspore yield and embryogenesis frequency is clear. For instance, in radish (*Raphanus sativus* ‘Taebaek’), approximately 10 × 10^4^ microspores per bud were isolated from 4 mm buds, which was significantly higher than that obtained from other bud sizes, and embryo production under this condition was approximately fivefold higher [28]. Similarly, in broccoli, 4–6 mm buds yielded a higher number of microspores per bud and produced more embryos than 2–4 mm [29]. This comparison highlights the importance of bud size in achieving optimal microspore yield and embryogenic responses across different *Brassica* species and related crops. Further studies could explore the underlying biological mechanisms that link bud size to microspore development and embryogenesis potential.

In general, the initial phase of heat shock induces rapid expression of heat shock proteins (HSPs), which are required to trigger the reprogramming of isolated microspores from the gametophytic to the sporophytic developmental pathway, thereby initiating embryogenesis [30]. Beyond this initial trigger, heat stress has also been reported to promote broader cellular changes, including alterations in hormonal balance, auxin biosynthesis and signaling, as well as chromatin remodeling, which are closely linked to embryogenic competence [31]. However, prolonged exposure to elevated temperatures leads to the accumulation of cellular damage and consequently reduces embryogenesis efficiency [32,33]. Accordingly, most previous studies have reported a decline in embryogenesis with increasing duration of heat treatment. Takahata and Keller (1991) [34] reported that, in microspore cultures of *Brassica oleracea* L., at 32.5 °C for 48 h or longer inhibited embryogenesis compared with a 24 h. Similarly, da Silva Dias (2001) [35] demonstrated that a heat shock of 32.5 °C for 24 h was optimal for embryogenesis in several broccoli genotypes, whereas extending the treatment duration resulted in reduced responsiveness. Similarly, embryogenesis was observed in green cabbage ‘SJ-Ca 13’ at 32.5 °C for 24 and 48 h, whereas it was completely inhibited after 72 h. In contrast, in cabbage microspore cultures, treatment at 30 °C for 48 h has been reported to maximize microspore embryogenesis in certain genotypes [25]. These findings suggest that optimal heat shock conditions for microspore embryogenesis may vary depending on genotype and that high-temperature, short-duration treatments are not necessarily the most effective. Therefore, optimization studies considering a wider range of temperatures and treatment durations are required to maximize microspore embryogenesis efficiency.

Agar concentration in the regeneration medium is an important physical parameter influencing plant regeneration efficiency from microspore-derived embryos. Low agar concentrations increase water availability in the medium, which can promote hyperhydricity and inhibit normal organ differentiation [36]. Previous studies have shown that relatively high agar concentrations can enhance regeneration efficiency. For example, in rapeseed, a relatively high agar concentration (1.6%) has been reported to enhance regeneration efficiency [37]. Peng et al. (1994) [38] compared different agar concentrations and reported that the optimal matric potential for plant regeneration from microspore-derived embryos ranged from −3 to −4 kPa, a condition typically achieved under higher agar concentrations. In this study, increasing the agar concentration in the regeneration medium from 0.9% to 1.0% resulted in an approximately 1.75-fold increase in the regeneration efficiency of microspore-derived embryos in green cabbage ‘SJ-Ca 13’. In addition to imposing moderate osmotic stress, higher agar concentrations may also influence regeneration by modulating ion availability, reducing the diffusion of nutrients, and limiting the rapid dispersal of embryo-derived metabolites, thereby creating a more conditioned microenvironment around developing embryos. Overall, these results suggest that moderate osmotic stress together with changes in the physicochemical properties of the medium, directly promotes plant regeneration.

The strength of MS salts is also a critical factor influencing plant regeneration. MS basal medium contains relatively high levels of nitrogen sources (NH_4_^+^/NO_3_^−^), calcium, sulfate (SO_4_^2−^), and micronutrients, which can support embryo maturation and meristem formation. However, it is not optimal for all genotypes. An increased NH_4_^+^/NO_3_^−^ ratio has been reported to enhance osmotic stress, ammonium toxicity, and the accumulation of reactive oxygen species (ROS), which in some Brassicaceae genotypes may promote callus formation or abnormal embryo development rather than shoot regeneration [39,40]. These findings support the notion that, in immature (heterotrophic) tissues such as microspore-derived embryos, a moderately reduced salt strength is more conducive to the maintenance of the shoot apical meristem and subsequent shoot regeneration than excessively high salt strength. In this study, all embryos failed to survive in full-strength (1×) MS medium, whereas successful shoot regeneration was observed on reduced salt media (1/2× and 1/3× MS). Similarly, previous studies have reported that microspore-derived embryos of broccoli [41], cabbage [42] and kale [43] regenerate efficiently on 1/2× MS medium, further suggesting that reduced MS salt strength can be favorable for regeneration from microspore-derived embryos in certain species or genotypes.

In microspore-derived embryos, adventitious shoot development may occur when embryogenic processes, including the establishment of apical–basal and radial polarity, differentiation of the primary tissue layers (epidermis, cortex, and endodermis), and proper formation of the shoot apical meristem (SAM), are not correctly completed [44,45]. Such defects can result in abnormalities at the shoot pole, including cotyledon fusion and the absence or dysfunction of the SAM. Under normal embryo development, the establishment of a functional SAM typically leads to the formation of a single shoot from a single apical region. However, in this study, cabbage microspore-derived embryos frequently produced adventitious shoots. Similar phenomena have been reported in microspore cultures of red cabbage, where shoots were induced from non-SAM regions such as the hypocotyl or cotyledons [46]. In rapeseed, adventitious shoots have also been observed to originate from enlarged embryos [47]. These observations suggest that disruption of the normal embryonic axis in microspore-derived embryos may lead to the formation of ectopic meristematic regions, resulting in shoot initiation at non-apical positions [48].

Previous studies have reported relatively low frequencies of spontaneous chromosome doubling in microspore cultures of oat and pepper, with rates of approximately 6% and 25%, respectively [49,50]. In contrast, high frequencies of spontaneous chromosome doubling have been reported in Brassicaceae crops, including Chinese cabbage (70.5–74.3%), cabbage (21–67%), and broccoli (52–100%) [51,52,53]. In this study, ploidy analysis of microspore-derived cabbage plants using flow cytometry showed that 50% of the regenerated plants were spontaneous doubled haploids. Such a high frequency of spontaneous chromosome doubling is consistent with previous observations in Brassicaceae species. This characteristic is particularly advantageous for breeding purposes, as it allows the efficient production of doubled haploid lines without the need for artificial chromosome doubling treatments, such as colchicine or oryzalin.

In *Brassica* crops, a wide range of molecular marker systems has been used to analyze genetic variation and traits related to disease resistance. Low- to medium-density markers such as RAPD and SSR have been effectively used to confirm genetic uniformity, gametophytic origin, and homozygosity in doubled haploid populations [54,55]. However, several studies have demonstrated that higher-resolution marker systems are required for fine-scale discrimination among closely related regenerants or for dissecting complex traits associated with microspore embryogenesis [56,57].

In this study, analysis using 26 SSR markers was not sufficient to reliably distinguish individual regenerants or to fully verify genetic variation. This limitation likely reflects the restricted number and genomic coverage of the SSR markers employed. Therefore, the application of genome-wide marker systems such as SNP arrays or genotyping-by-sequencing would be required for more robust validation of genetic variation in Brassicaceae microspore-derived doubled haploid populations.

Further self-pollination and stabilization of the regenerated plants are required to enable the evaluation of horticultural traits of DH lines. The identification and selection of DH lines would facilitate their utilization as valuable breeding materials and could contribute to the development of improved F_1_ hybrids.

## 4. Materials and Methods

### 4.1. Plant Material and Donor Plant Growth

Cabbage ‘SJ-Ca 13’, a green cabbage (*Brassica oleracea* var. *capitata)* characterized by compact head density, small head size, clubroot resistance, and a low core ratio, was used as the donor plant in this study, and seeds were obtained from Heungnong Seed Co., Ltd., Sejong, Republic of Korea.

Seeds were sown in August 2024. After bolting induced by exposure to low temperature (<4 °C) for approximately two months during winter, plants were transplanted in February 2025 into plastic pots and transferred to a growth room. Donor plants were then maintained for two weeks under controlled conditions with a 16 h light/8 h dark photoperiod at 20 °C/15 °C (Day/night) and a light intensity of approximately 250 µmol m^−2^ s^−1^. Flower buds were harvested after this acclimation period and used for subsequent microspore culture experiments.

### 4.2. Microspore Isolation and Culture

Microspore isolation and culture were performed using a modified protocol described by Kozar et al. (2022) [58]. Flower buds of three different sizes (2 ± 0.5, 4 ± 0.5, and 6 ± 0.5 mm) were collected from the entire inflorescence, and microspores isolated from each bud size were cytologically examined to confirm their developmental stages before further treatments. Thirty flower buds of three different sizes were placed in conical tubes containing 20 mL of water, and cold-pretreated at 4 °C for 24 h. The conical tube containing cold pretreated buds was transferred to a laminar flow hood. Finally, buds were sterilized with 20 mL of 1.5% sodium hypochlorite solution under vacuum for 15 min. After vacuum release, the buds were rinsed three times with sterile distilled water for 3 min. Surface-sterilized floral buds were cut in half and placed in a 15 mL conical tube containing 7 mL of NLN-13 liquid medium (Nitsch and Nitsch; 13% sucrose, pH 5.8; KisanBio, Seoul, Republic of Korea) [59] and were vortexed for 1 min to facilitate the release of microspores. The macerate was filtered through a 45 µm sieve. The filtrate was adjusted to 25 mL with NLN-13 liquid medium and centrifuged at 1000 rpm for 3 min at 4 °C using a swing-out rotor (Model 1736R; Labogene, Lillerød, Denmark). After discarding the supernatant, the pellet was washed twice under the same conditions with NLN-13 medium. The pellet was resuspended in 10 mL of NLN-13 liquid medium, and microspore density was adjusted to 4 × 10^4^ microspores/mL based on hemocytometer counts (Incyto, Cheonan-si, Republic of Korea). The number of isolated microspores per bud was determined using a hemocytometer. 5 mL of microspore suspension was plated in 60 × 15 mm Petri dishes. Petri dishes were incubated at 32.5 °C in darkness for 0 h, 24 h, 48 h, 72 h to induce microspore embryogenesis, and then at 25 °C until visible embryos appeared. Subsequently, the cultures were placed on a shaker (Hangil, Seoul, Republic of Korea) at 70 rpm under light conditions (25 °C, approximately 40 µmol m^−2^ s^−1^, 16/8 h light/dark photoperiod). Four weeks after microspore isolation, the number of embryos per Petri dish was recorded. Morphological development of microspore-derived embryos was examined using a light microscope (Model OAM-24NS; Dongwon CNS, Seongnam, Republic of Korea). Each treatment was performed with three replicates (200,000 microspores per plate). All experiments were repeated independently three times under the same conditions.

### 4.3. Plant Regeneration

Embryos larger than 0.5 cm which were derived from microspores were transferred to semi-solid MS (Murashige and Skoog; KisanBio, Seoul, Republic of Korea) medium [60] with different salt strengths (1×, 1/2×, and 1/3×) and agar concentrations (0.9% and 1.0%; Samchun, Seoul, Republic of Korea), supplemented with 3% sucrose and adjusted to pH 5.8. Five embryos were placed in each glass culture vessel, with five vessels per treatment. All cultures were maintained for two months at 25 °C with 16 h light and 8 h dark photoperiod with light intensity of approximately 40 µmol m^−2^ s^−1^. The percentage of shoot regeneration and the number of shoots per embryo were recorded after two months of culture. The regenerated shoots were then subcultured onto medium to form complete plantlets.

### 4.4. Acclimatization

One week prior to transplantation, regenerated plantlets were acclimatized by increasing light intensity from approximately 40 to 80 µmol m^−2^ s^−1^ and partially opening the lids of culture vessels to gradually reduce internal humidity. At transplantation, residual medium on the roots of the plantlets was gently washed off with lukewarm water, and the plantlets were transplanted individually into plastic cup pots (10 cm diameter × 9 cm height × 7 cm base diameter) containing a sterilized perlite:vermiculite mixture (2:1, *v*/*v*; Myplant, Yangsan, Republic of Korea).

Transplanted plantlets were covered with transparent polyethylene bags and maintained at 25 °C under a 16 h light/8 h dark photoperiod with a light intensity of approximately 80 µmol m^−2^ s^−1^. Two weeks after transplantation, the upper part of the bags was gradually opened to reduce humidity, and the bags were completely removed one week later. Two weeks after bag removal, the potting substrate was replaced with 100% commercial horticultural soil (Baroker, Eumseong, Republic of Korea) to promote development (Figure 7).

### 4.5. Ploidy Level Determination

Ploidy level of regenerated plants was determined following the protocol described by Ahn et al. [61] using a flow cytometer (Sysmex Partec GmbH, Goerlitz, Germany). DNA content was measured relative to a diploid cabbage (donor plant) standard, and ploidy levels were determined by comparison of fluorescence peak. Fluorescence peaks at approximately 100, 200, and 400 were interpreted as haploid, diploid (doubled haploid), and tetraploid levels, respectively [9].

After ploidy determination, regenerated plants were grown for two months under the same growth room conditions described above, and plant width and leaf length were measured. Regenerated plants were subsequently maintained under low-temperature conditions (<4 °C) for eight weeks for vernalization. Morphological differences in floral traits among plants with different ploidy levels were observed.

The absence of haploid data is due to the lack of bolting and flowering in haploid plants. In addition, haploid plants were not included in the marker analysis because they did not flower and were therefore not practically applicable.

### 4.6. SSR Marker Analysis of Regenerated Plants from Microspore-Derived Embryos

Young leaves were collected from the donor plant and from microspore-derived plants after regeneration and acclimatization. Genomic DNA was extracted using a modified phenol–CTAB method as described previously [62].

PCR amplification was performed in a total reaction volume of 15 µL containing 7.5 µL Dyne Ready 2× GO Taq Mix (Star Plus Taq with Dye; Dynebio Co., Gyeonggido, Republic of Korea), 1.5 µL 5× Tune-Up Solution, 1.5 µL each of forward and reverse primers (1 µM), 1.5 µL template DNA (50 ng/µL), and 1.5 µL distilled water [63].

PCR conditions consisted of denaturation at 95 °C for 7 min, followed by 35 cycles of denaturation at 94 °C for 10 s, annealing at 56 °C for 30 s, and extension at 72 °C for 30 s, with a final extension at 72 °C for 7 min.

A total of 26 SSR primer sets commonly used for cabbage purity testing in our laboratory were employed [64,65,66], and primer information is provided in Supplementary Table S1. PCR products were separated on 3% agarose gels prepared in 0.5× TBE buffer, and the gels were stained with EcoDye (Biofact Co., Daejeon, Republic of Korea). Electrophoresis was conducted at 100 V for 15 min followed by 180 V for 60 min. DNA bands were visualized under UV illumination, and a 100 bp DNA ladder was used as the size marker.

### 4.7. Statistical Analysis

Statistical analysis was performed using SPSS Statistics 27 software (IBM Corp., Armonk, NY, USA), with mean comparisons assessed by Duncan’s multiple range test. Statistical significance was determined at *p* < 0.05.

## 5. Conclusions

In this study, an efficient isolated microspore culture system for green cabbage was established to support doubled haploid production. Key factors affecting microspore embryogenesis and plant regeneration, including flower bud size, heat shock treatment, and regeneration medium composition, were systematically evaluated. The optimized conditions identified in this study provide a practical basis for improving the efficiency of doubled haploid line development in cabbage breeding programs.

## Figures and Tables

**Table 1:** Effect of bud size on embryogenesis of microspore culture in green cabbage ‘SJ-Ca 13’.

Donor Plant	Bud Size (mm)		
	2 ± 0.5	4 ± 0.5	6 ± 0.5
SJ-Ca 13	0.0 ± 0.00 ^b^	2.33 ± 0.57 ^a^	0.0 ± 0.00 ^b^

Values are the mean number of embryos per Petri dish ± SD. Values within the row followed by different letters are significantly different at the 95% level by Duncan’s test.

**Table 2:** Effect of heat shock duration on embryogenesis of microspore culture in green cabbage ‘SJ-Ca 13’.

Donor Plant	Duration of Heat Shock (h)			
	0	24	48	72
SJ-Ca 13	0.0 ± 0.00 ^b^	2.33 ± 0.52 ^a^	2.00 ± 0.82 ^a^	0.0 ± 0.00 ^b^

Values are the mean number of embryos per Petri dish ± SD. Values within the row followed by different letters are significantly different at the 95% level by Duncan’s test.

**Table 3:** Adventitious shoot regeneration rate and shoot number per microspore-derived embryo cultured on different MS salt strengths and agar concentrations in green cabbage ‘SJ-Ca 13’.

MS Strength	Agar Concentration	Shoot Regeneration (%)	Number of Shoots per Embryo
1/3×	0.9%	16.00 ± 8.94 ^ab^	2.00 ± 1.41 ^ab^
1/3×	1.0%	28.00 ± 17.88 ^a^	2.40 ± 1.51 ^a^
1/2×	0.9%	0.00 ± 0.00 ^c^	0.00 ± 0.00 ^b^
1/2×	1.0%	8.00 ± 4.47 ^bc^	2.00 ± 0.75 ^ab^
1×	0.9%	0.00 ± 0.00 ^c^	0.00 ± 0.00 ^b^
1×	1.0%	0.00 ± 0.00 ^c^	0.00 ± 0.00 ^b^

The different letters within the same column show significant differences at *p* < 0.05 (DMRT).

**Table 4:** Ploidy identification of regenerated plants from microspore-derived embryos in green cabbage ‘SJ-Ca 13’.

No. of Evaluated Regenerated Plants	Number of Regenerated Plants by Ploidy Level (%)		
	Haploid	Spontaneous Doubled Haploid	Tetraploid
12	3 (25)	6 (50)	3 (25)

**Table 5:** Morphological characteristics of regenerated plants from microspore-derived embryos with different ploidy levels in green cabbage ‘SJ-Ca 13’ after two months of acclimatization.

Ploidy	Plant Width (cm)	Leaf Length (cm)
Control (2n)	31.33 ± 1.15 ^a^	17.33 ± 1.52 ^b^
Haploid	26.33 ± 5.50 ^a^	12.66 ± 2.08 ^b^
Spontaneous doubled haploid	29.00 ± 3.60 ^a^	14.00 ± 1.00 ^b^
Tetraploid	30.33 ± 3.47 ^a^	16.67 ± 3.05 ^b^

The different letters within the same column show significant differences at *p* < 0.05 (DMRT).

## Data Availability

The original contributions presented in this study are included in the article/Supplementary Material. Further inquiries can be directed to the corresponding author.

## Supplementary Materials

The following supporting information can be downloaded at https://www.mdpi.com/article/10.3390/plants15020221/s1. Table S1: Information of 26 SSR markers primer sets.
