# Supplementary material for: Induction and Regeneration of Microspore-Derived Embryos for Doubled Haploid Production in Cabbage (Brassica oleracea var. capitata)
Source: Plants (Basel). 2026 Jan 10;15(2):221. doi: 10.3390/plants15020221 (PMC12845209; doi:10.3390/plants15020221)
Supplement: Supplementary file 1 [file plants-15-00221-s001.zip › Supplementary Table S1.pdf]

Supplementary Table S1. Information of 26 SSR markers primer sets

Primer name	Forward /Reverse	Primer sequence (5' to 3')
BoL-1	F	GCCTTTCTTCACAACTGATAGCTAA
	R	TCAGGTGCCTCGTTGAGTTC
BoL-2	F	ATTACAAAAATGCCCTGAC
	R	TAAGTGATCTTCTCTCCAACA
BoL-3	F	CTCGATCTTCCCCTGCTTTC
	R	GTTGAGCCAATCTACGGTTCC
BoL-4	F	GGATCAGTTATCTGCACCACAA
	R	TCGGAATTGGATAAGAATTCAA
BoL-5	F	CAAAGCGAGAAAGTGCAGTTGAGAG
	R	TCCACGAAACTACTGCAGATTGAAA
BoL-6	F	CATCCTAATGTTGCTGAGAAAGAGG
	R	TATATGAAACCGATGAAGCTCCTTT
BoL-7	F	AAGTCGTTAGGCGAATCTG
	R	ATTGAAGAGGAAGAAGGAGAA
BoL-8	F	GCACACTCCACTACACGAA
	R	ACCATTCAACCACTCAAATC
BoL-9	F	AGCAATAAGCCAGAAACTTG
	R	GTTCATCATCACAACTCTAACCT
BoL-10	F	TGAAGATGGGACTCAAACA
	R	GATGAAGCAGAGAATGACAAG
BoL-11	F	TGTTTTGATGTTTCCTACTG
	R	GAACCTGTGGCTTTTATTAC
BoL-12	F	AAGAACGTCAAGATCCTCTGC
	R	ACCACCACGGTAGTAGAGCG
BoL-13	F	ATCGTTGCCATTAGGAGTGG
	R	ACCAAATTAACCCTCTTTGC
BoL-14	F	GCAAACGATTTGTTTACCCG
	R	CGTGTAGGGTGATCTAGATGGG
BoL-15	F	GGCGACATAGATTTGAACCG
	R	TCCACTTTCTCTCTCTTCCCC
BoL-16	F	GAGTGACATCGAAAATCAGATAGC
	R	CCTAAATGGAAAGGCTTGGC
BoL-17	F	ACATTCTTGGATCTTGATTCG
	R	AAAGGTCAAGTCCTTCCTTCG
BoL-18	F	CCTCTTCAGTCGAGGTCTGG
	R	AATTTGGAAACAGAGTCGCC
BoL-19	F	TCCGAACACTCTAAGTTAGCTCC
	R	GAGCTGTATGTCTCCCGTGC
BoL-20	F	TCGCGACGTTGTTTTGTTC
	R	ACCATCTTCCTCGACCCTG
BoL-21	F	TCCGAACACTCTAAGTTAGCTCC
	R	TTCTTCACTTCACAGGCACG
BoL-22	F	TGGGTAAGTAACTGTGGTGGC
	R	AGAGTTCGCATACTCTGGAGC
BoL-23	F	CGAACATCTTAGGCCGAATC
	R	GGTTAACCTGCGGGATATTG
BoL-24	F	TGTCAGTGTGTCCACTTCGC
	R	AAGAGAAACCCAATAAAGTAGAACC
BoL-25	F	ACAGCAAGGATGTGTTGACG
	R	GATGAGCCTCTGGTTCAAGC
BoL-26	F	CCCCTTCCGGTTAAACAAAT
	R	AAAACAGACTTTGCCCGTTG
